# H_2_O_2_ attenuates IGF-1R tyrosine phosphorylation and its survival signaling properties in neuronal cells via NR2B containing NMDA receptor

**DOI:** 10.18632/oncotarget.18625

**Published:** 2017-06-27

**Authors:** Zhiwen Zeng, Dejun Wang, Uma Gaur, Liao Rifang, Haitao Wang, Wenhua Zheng

**Affiliations:** ^1^ Faculty of Health Sciences, University of Macau, Taipa, China; ^2^ Shenzhen Mental Health Center and Shenzhen Kangning Hospital, Shenzhen, China; ^3^ Department of Pharmacy, Qingdao Huangdao District People’s Hospital, Qingdao, China

**Keywords:** H_2_O_2_, SH-SY5Y, NR2B, NMDA, IGF-1R

## Abstract

Impairment of insulin-like growth factor I (IGF-I) signaling plays an important role in the development of neurodegeneration. In the present study, we investigated the effect of H_2_O_2_ on the survival signaling of IGF-1 and its underlying mechanisms in human neuronal cells SH-SY5Y. Our results showed that IGF-1 promoted cell survival and stimulated phosphorylation of IGF-1R as well as its downstream targets like AKT and ERK1/2 in these cells. Meanwhile, these effects of IGF-1 were abolished by H_2_O_2_ at 200μM concentration which did not cause any significant toxicity to cells itself in our experiments. Moreover, studies using various glutamate receptor subtype antagonists displayed that N-methyl-D -aspartate (NMDA) receptor antagonist dizocilpine maleate (MK-801) blocked the effects of H_2_O_2_, whereas other glutamate receptor subtype antagonists, such as non-NMDA receptor antagonist 6,7-dinitroquinoxaline-2,3-dione (DNQX), metabolic glutamate receptor antagonists LY341495 and CPCCOEt, had no effect. Further studies revealed that NR2B-containing NMDARs are responsible for these effects as its effects were blocked by pharmacological inhibitor Ro25-698 or specific siRNA for NR2B, but not NR2A. Finally, our data also showed that Ca^2+^ influx contributes to the effects of H_2_O_2_. Similar results were obtained in primary cultured cortical neurons. Taken together, the results from the present study suggested that H_2_O_2_ attenuated IGF-1R tyrosine phosphorylation and its survival signaling properties via NR2B containing NMDA receptors and Ca^2+^ influx in SH-SY5Y cells. Therefore, NMDAR antagonists, especially NR2B-selective ones, combined with IGF-1 may serve as an alternative therapeutic agent for oxidative stress related neurodegenerative disease.

## INTRODUCTION

Progressive oxidative stress is a major event which leads to neuronal death in various neurodegenerative diseases, including Alzheimer’s, Parkinson’s and Huntington’s disease [1–3]. Although oxidative stress lacks any specific clinical symptom, yet it is observed that reactive oxygen species (ROS) or/and reactive nitrogen species (RNS) are its main causative agents [4, 5]. Under balanced conditions, cell components are protected by antioxidants from oxidative damage by converting H_2_O_2_ into O_2_ and H_2_O; whereas exaggerated ROS levels oppress the antioxidant capacity and consequently the cell suffers insult [4, 6]. Conventionally, H_2_O_2_ has been used to induce oxidative stress in cultured cells [7, 8].

IGF-1 is a polypeptide growth factor having similar structure and function as insulin and performs multiple crucial functions in the Central Nervous System (CNS) [9]. Changes in IGF-1 signaling have been appeared to be linked with cognitive deterioration in AD patients [10, 11], and in mouse model of AD the IGF-1 administration recovered the cognitive functioning [12]. The biological activities of IGF-1 are mainly arbitrated by type I IGF receptors [13]. IGF-1 binds to these receptors which initiates its auto phosphorylation, which further triggers its intrinsic tyrosine kinases, resulting in the phosphorylation of many intracellular substrates, followed by activation of various downstream signaling pathways such as the phosphatidylinositol 3-kinase (PI3K)/AKT pathway and mitogen-activated protein kinase/extracellular signal-regulated kinase (MAPK/ERK1/2) [14]. IGF-1 confers neuroprotection via these signaling cascades against multiple abuses such as potassium deprivation, serum deprivation, SNP toxicity, cerebral ischemia, and glutamate excitotoxicity [15–18]. IGF-1 is gaining increasing attention as a potential therapeutic agent for the treatment of neurodegenerative disorders.

However, the therapeutic potential of IGF-1 may be affected by oxidative stress. For example, it has been reported that H_2_O_2_ treatment was able to induce extracellular accumulation of glutamate while glutamate and oxidative stress impaired IGF-1R signaling in cultured primary neurons [19, 20]. In the mammalian brain, Glutamate is the major excitatory neurotransmitter which helps in the transmission of basal excitatory synapses and generates various forms of synaptic plasticity associated with cognitive processes such as long term potentiation and long term depression [21]. Receptors belonging to two major subfamilies namely ionotropic and metabotropic receptors, mediate the effects of glutamate [22]. Glutamate have been shown to impair the IGF-1R signaling and its protective effects in cultured primary neurons in our previous report and the effects were mediated by NMDA receptors [19]. However, the effect of oxidative stress on IGF-1 signaling and its underlying mechanism in SH-SY5Y cells, a neuronal cellular model of human source for studying human neurodegenerative disease, is not known.

NMDARs are major glutamate receptors which are highly expressed in the CNS and play a crucial role in the action of glutamate, and also its excessive activation causes an increase in intracellular Ca^2+^ influx [23]. The increased Ca^2+^ is the main risk factor in neurodegenerative disorders which leads to neuron injury by apoptosis [24, 25]. NMDARs are heteromeric complexes made up of two obligatory NRI and two NR2A/2B/2C/2D subunits [26]. NR2B-containing NMDARs are the most important subunits related to excessive Ca^2+^ influx [27, 28].

The present study was designed with an aim to explore the neuroprotective effects of IGF-1, the inhibitory effects of H_2_O_2_ and the underlying mechanisms in SH-SY5Y cells. We reported here that IGF-1 protected SH-SY5Y cells from serum deprivation insult and H_2_O_2_ attenuated the survival promoting effects of IGF-1 by disturbing the IGF-1R survival signaling. The effect of H_2_O_2_ was mediated by the NMDA receptors as blocked by a specific antagonist, MK-801. Further study revealed that NR2B-containing NMDARs are responsible for these effects as these effects were abolished by pharmacological inhibitor or specific siRNA of NR2B. Finally, our data also displayed that Ca^2+^ influx contributes to the effects of H_2_O_2_. Taken together, these data revealed a novel mechanism whereby oxidative stress induced by H_2_O_2_ interferes trophic factor signaling linking to neurodegenerative diseases via NR2B containing NMDA receptors and Ca^2+^ influx in SH-SY5Y cells.

## RESULTS

### IGF-1 protected against serum deprivation insult and activated IGF-1R, AKT, ERK1/2 signaling in SH-SY5Y cells

**Figure 1:**
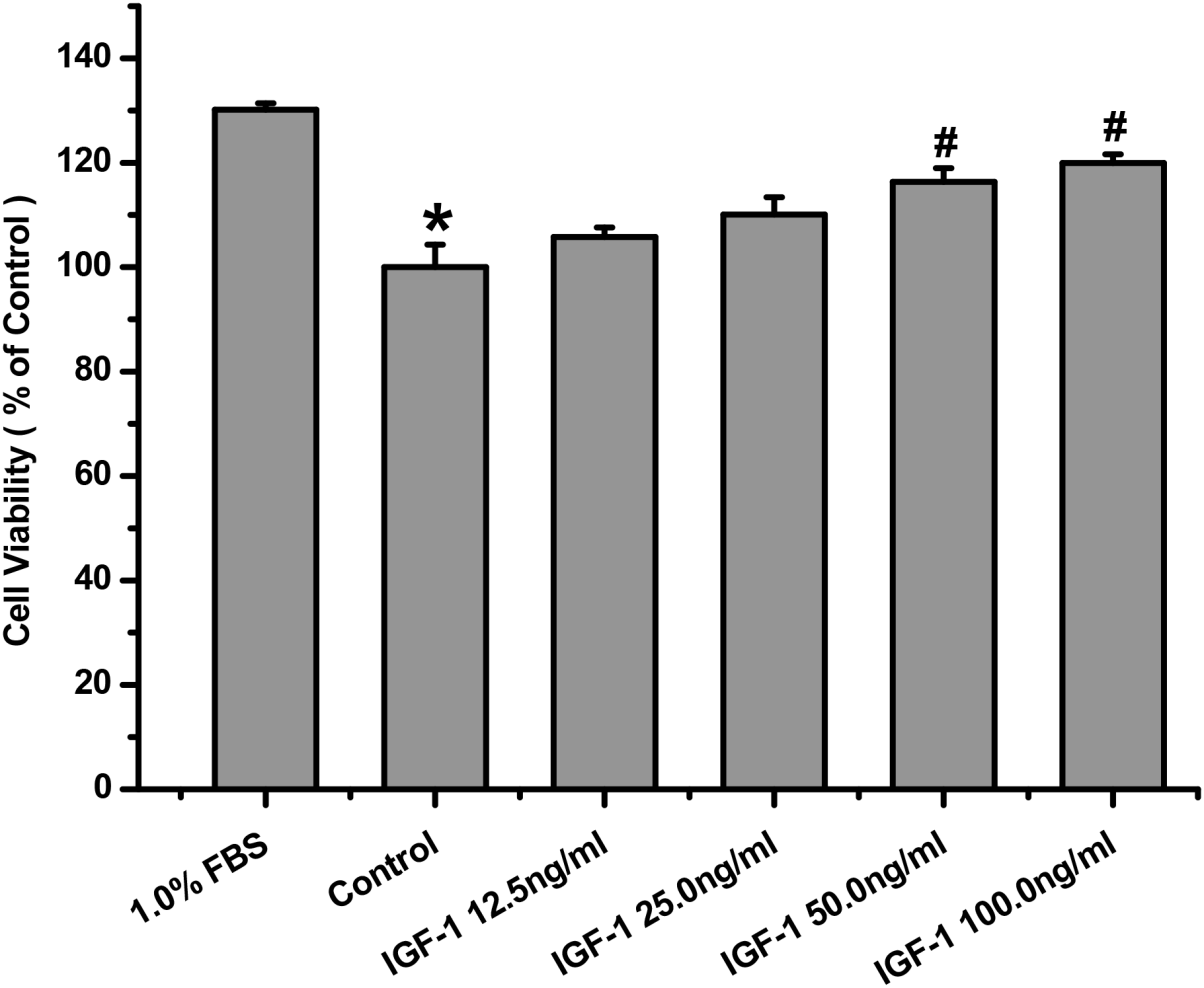

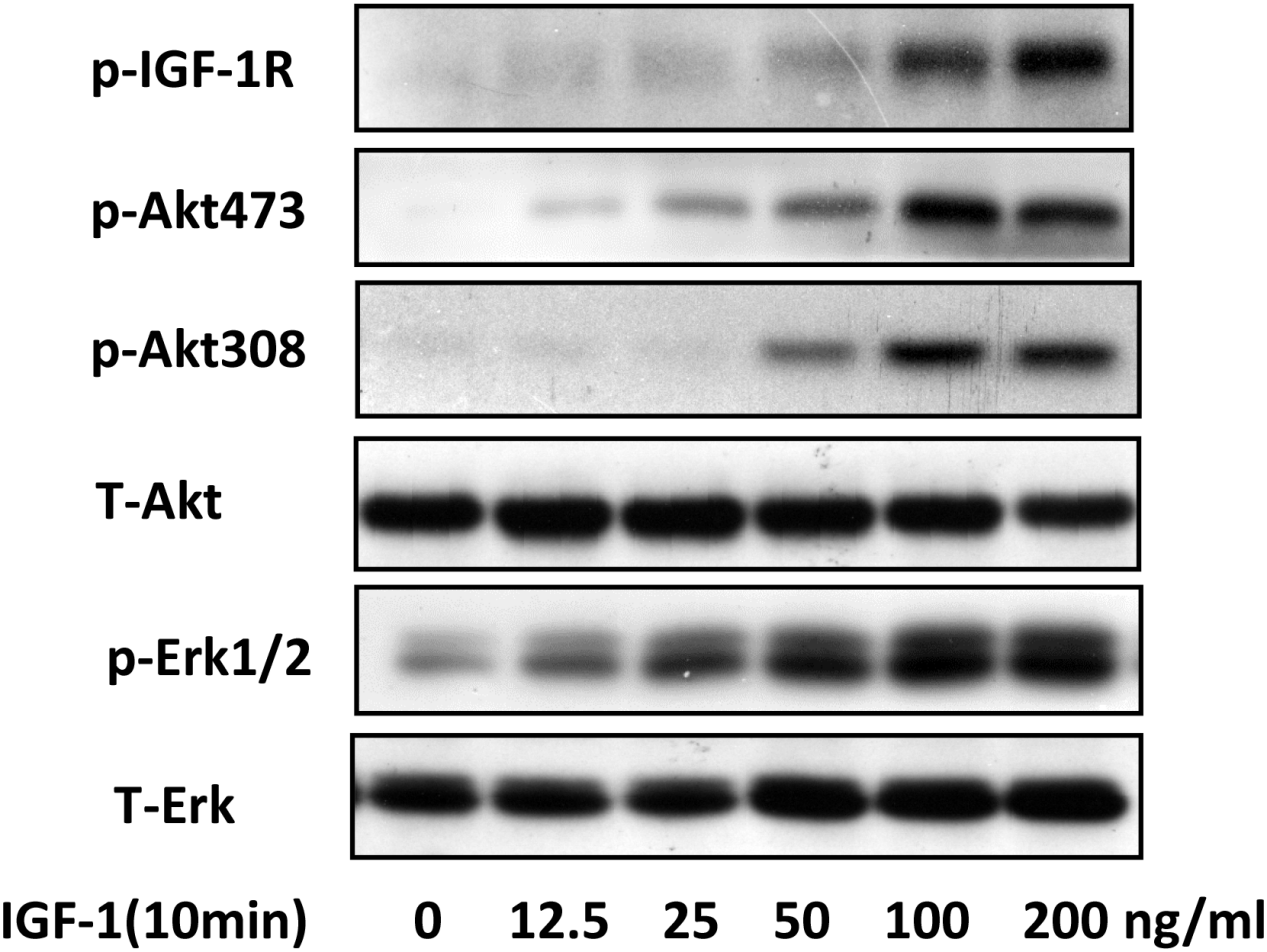

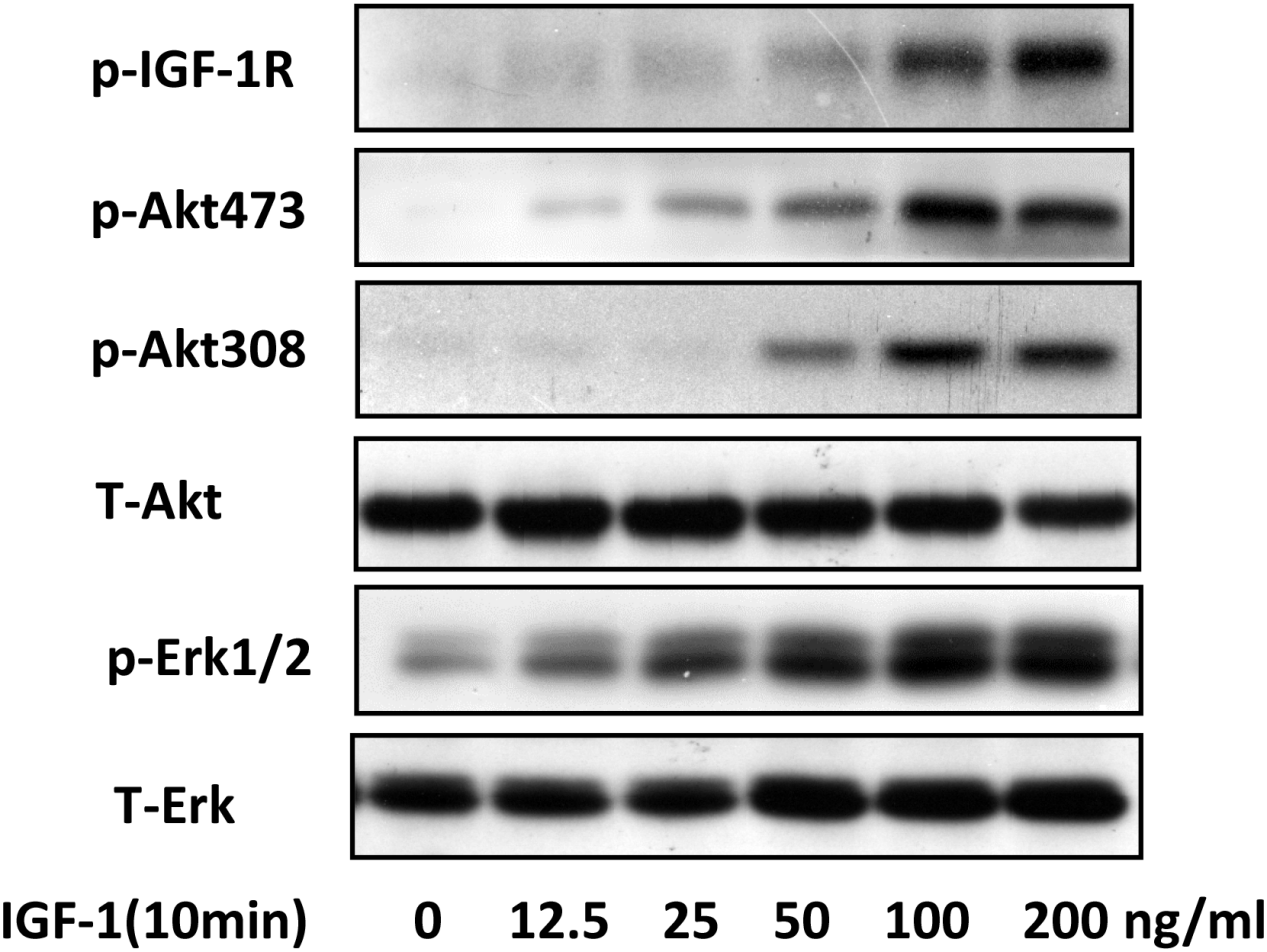

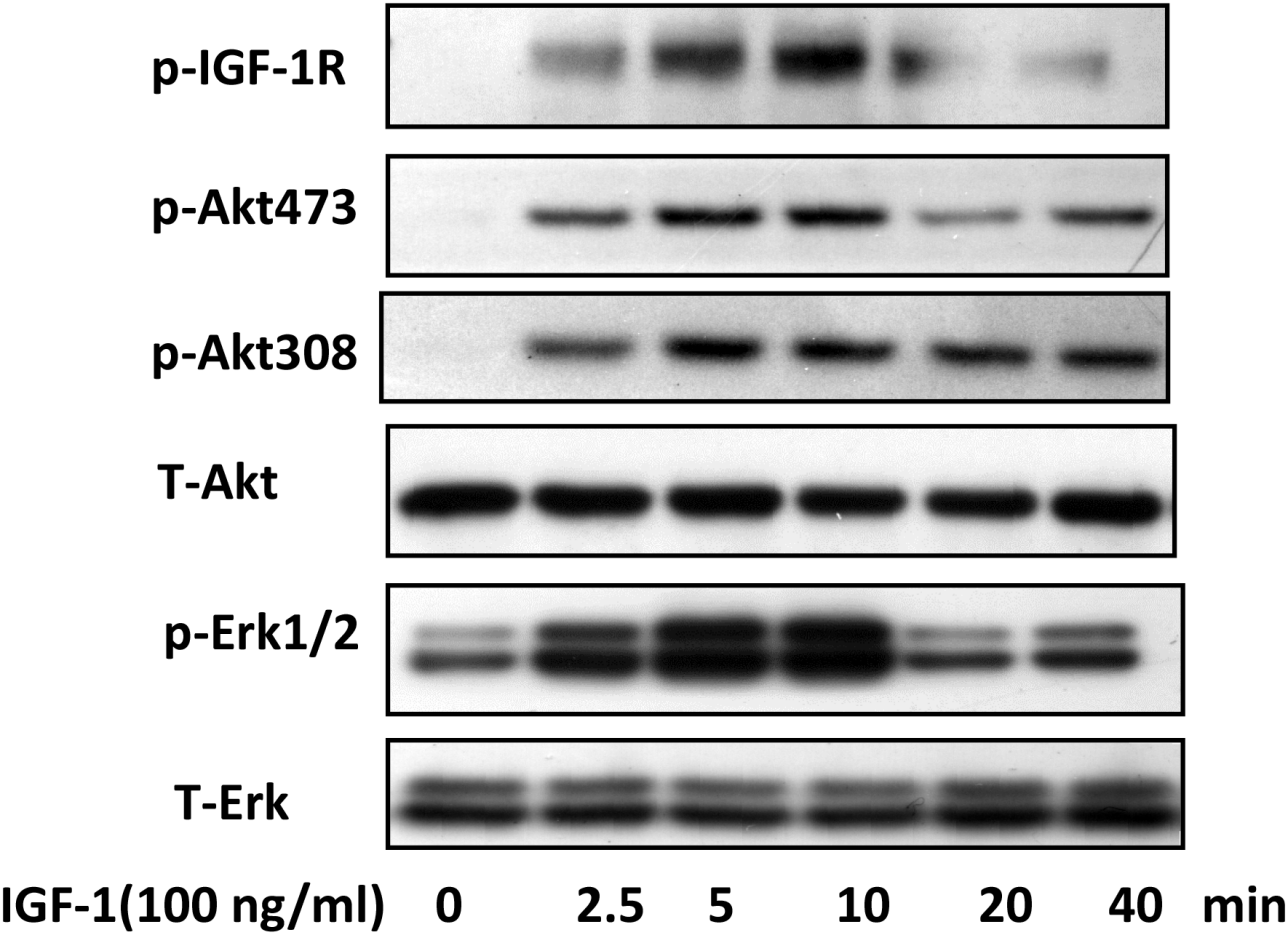
IGF-1 enhanced cell viability and activated IGF-1R, AKT, ERK1/2 signaling in SH-SY5Y cells **(a)** Serum deprived SH-SY5Y cells were exposed to various concentrations of IGF-1for 24 h. Cells viability was determined by MTT assay. *p <0.05 compared with serum 1%, #p <0.05 compared with control. **(b)** SH-SY5Y cells were treated with 100ng/ml IGF-1 for various times. **(c)** SH-SY5Y cells were treated with IGF-1 in different concentrations for 10min. Phosphorylation of IGF-1R, AKT and ERK1/2 was analyzed by western blot with specific antibodies as described in Material and Methods.

IGF-1 is a major survival promoting-trophic growth factor. We have previously reported its signaling and pro-survival effects in various cell lines but very less information is available about its survival promoting effects on SH-SY5Y cells. Here, we investigated the effects of IGF-1 treatment on the survival of SH-SY5Y cells by using the MTT assay. IGF-1 protected the SH-SY5Y cells against viability reduction caused by serum deprivation in a concentration-dependent manner (Figure 1a). As tyrosine phosphorylation of IGF-1R is the essential and initial step of IGF-1R signaling, we looked in to the tyrosine phosphorylation of IGF-1R and AKT/MAPK kinase, the key kinases in two major signaling pathways named the PI3K/AKT and the MAPK pathways involved in IGF-1 survival signaling. As expected, IGF-1 produced a stable and significant elevation of phosphorylated IGF-1R, AKT and ERK in a time- and concentration-dependent fashion in SH-SY5Y cells as shown in Figure 1b and 1c.

### H_2_O_2_ attenuates survival promoting effects and activation of IGF-1R, AKT, ERK1/2 signaling by IGF-1 in SH-SY5Y cells

**Figure 2:**
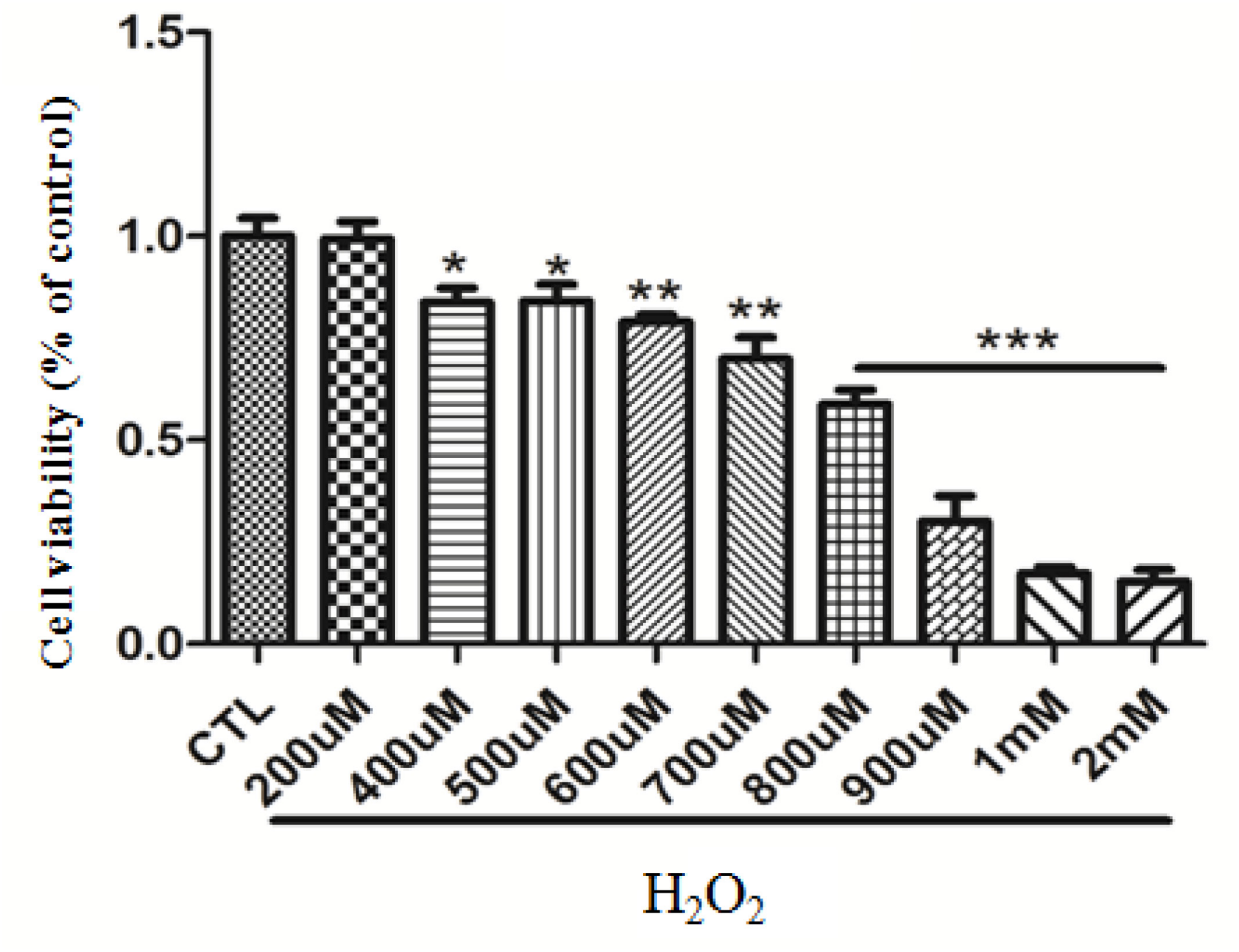

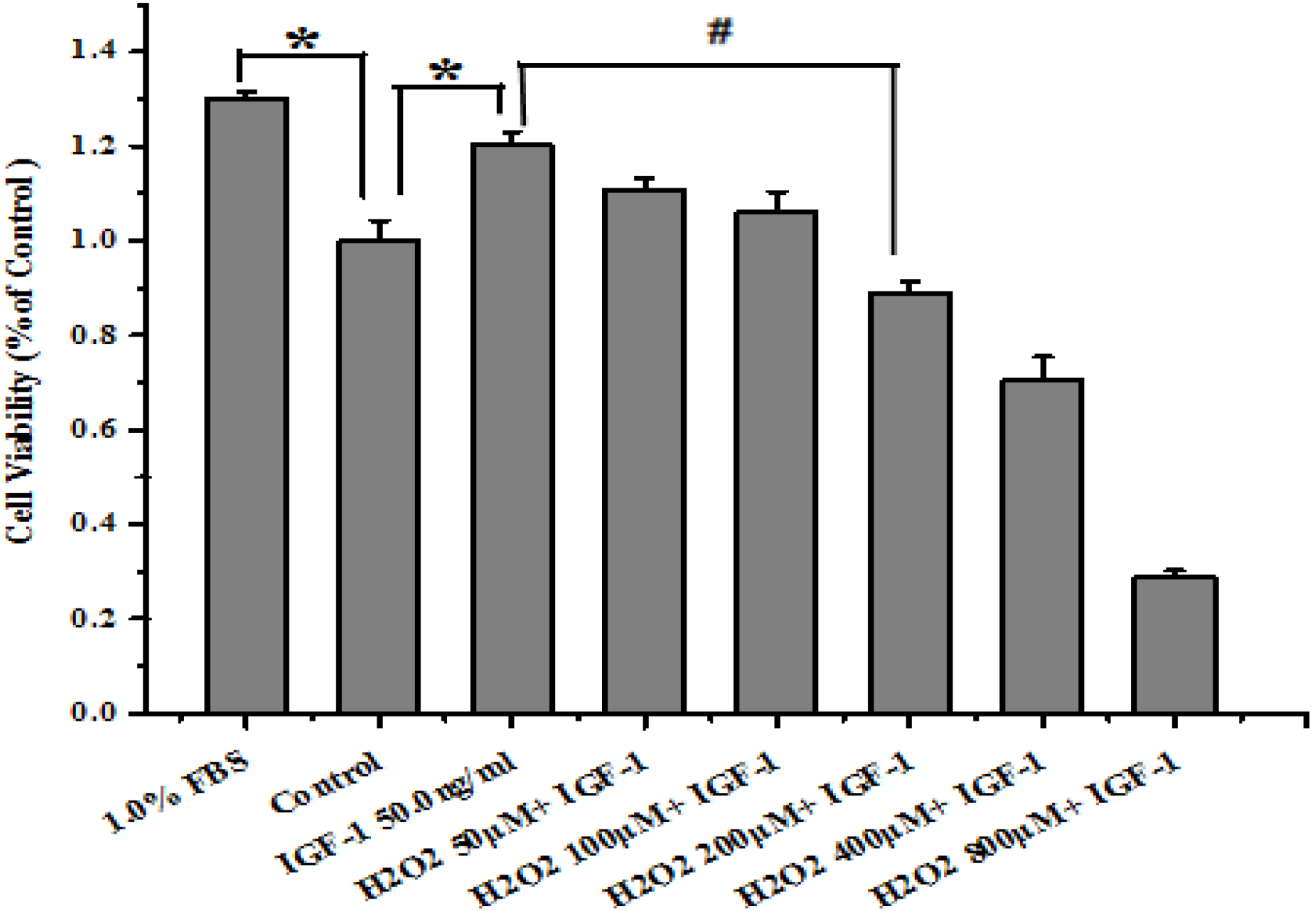

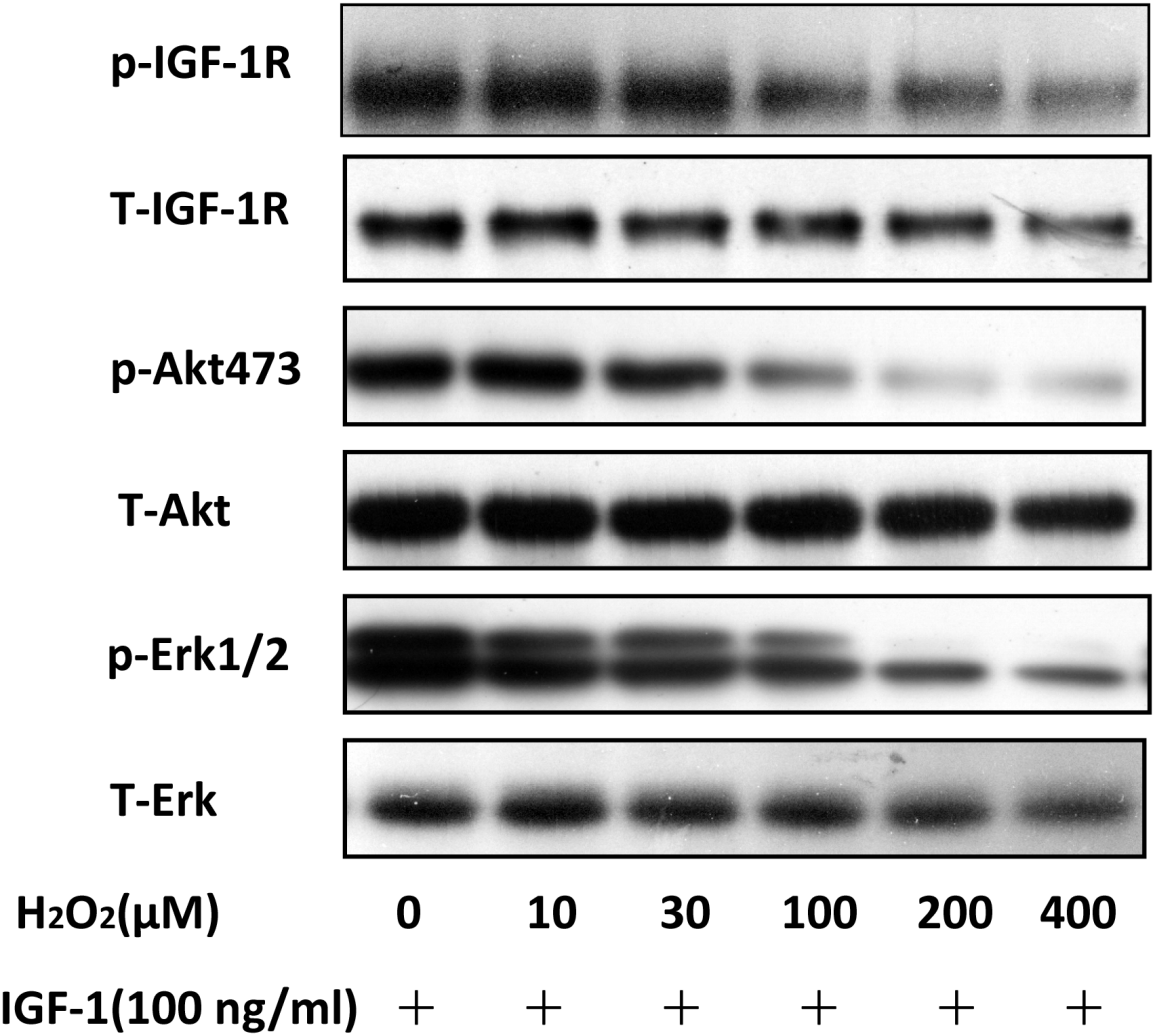
H_2_O_2_ attenuates survival promoting effects and phosphorylation of IGF-1R, AKT and ERK1/2 signaling stimulated by IGF-1 in SH-SY5Y cells **(a)** Cells were treated with various concentrations of H_2_O_2_ for 24h. Cells viability was determined by MTT assay *p <0.05 compared with control. **(b)** Cells were treated with various concentrations of H_2_O_2_ for 60 min alone or followed by stimulation with IGF-1, for 24h. Cell viability was determined by MTT assay *p <0.05 compared with 1% serum, #p <0.05 compared with control. **(c)** SH-SY5Y cells pretreated with different concentrations of H_2_O_2_ for 60min were incubated with 100ng/ml IGF-1 for10 min. Phosphorylation of IGF-1R, AKT and ERK1/2 was analyzed by western blotting with specific antibodies.

Firstly, we tested the toxicity of H_2_O_2._ As it can be seen in Figure 2a, H_2_O_2_ (200μM) itself had no toxic effects on SH-SY5Y cells. To go in to the effect of H_2_O_2_ on the survival promoting effects and signaling of IGF-1, cells were pretreated with different concentrations of H_2_O_2_ before stimulation with 50ng/ml IGF-1, and the cell viability and the phosphorylation levels of IGF-1R, AKT and ERK1/2 were measured by MTT and western blotting, respectively. As shown in Figure 2b, H_2_O_2_ attenuated survival promoting effects of IGF-1 in a concentration-dependent manner from 50μM and reached a statistical significance at 200μM which was also not significantly toxic to SH-SY5Y cells in our experiments. Meanwhile, H_2_O_2_ attenuated phosphorylation levels of IGF-1R, AKT and ERK1/2 induced by IGF-1 in a concentration dependent fashion and achieved a remarkable significance at 100-200μM (Figure 2c).

### MK-801 canceled the inhibitory effect of H_2_O_2_ on the pro-survival signaling and effects of IGF-1 in SH-SY5Y cells

**Figure 3:**
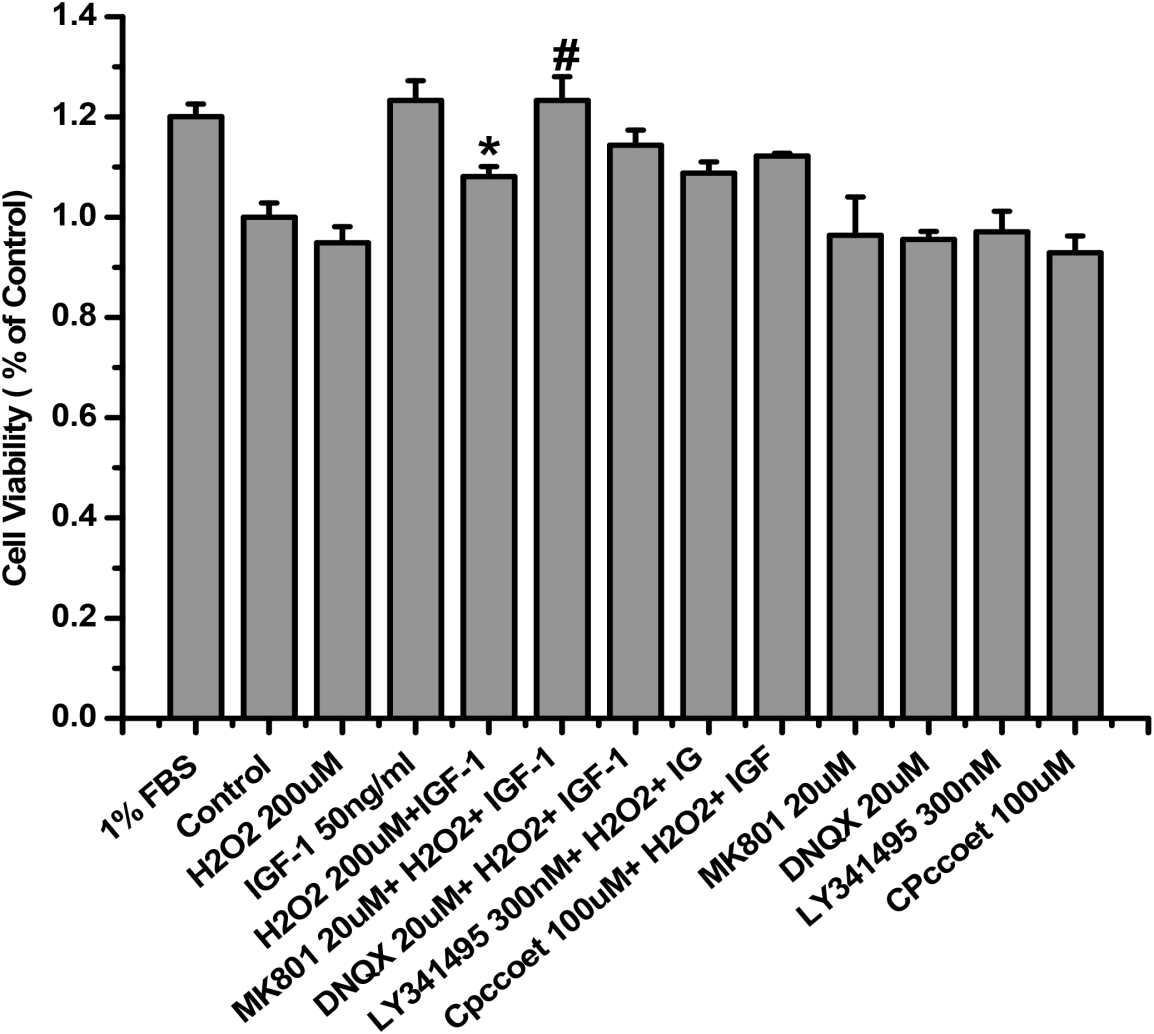

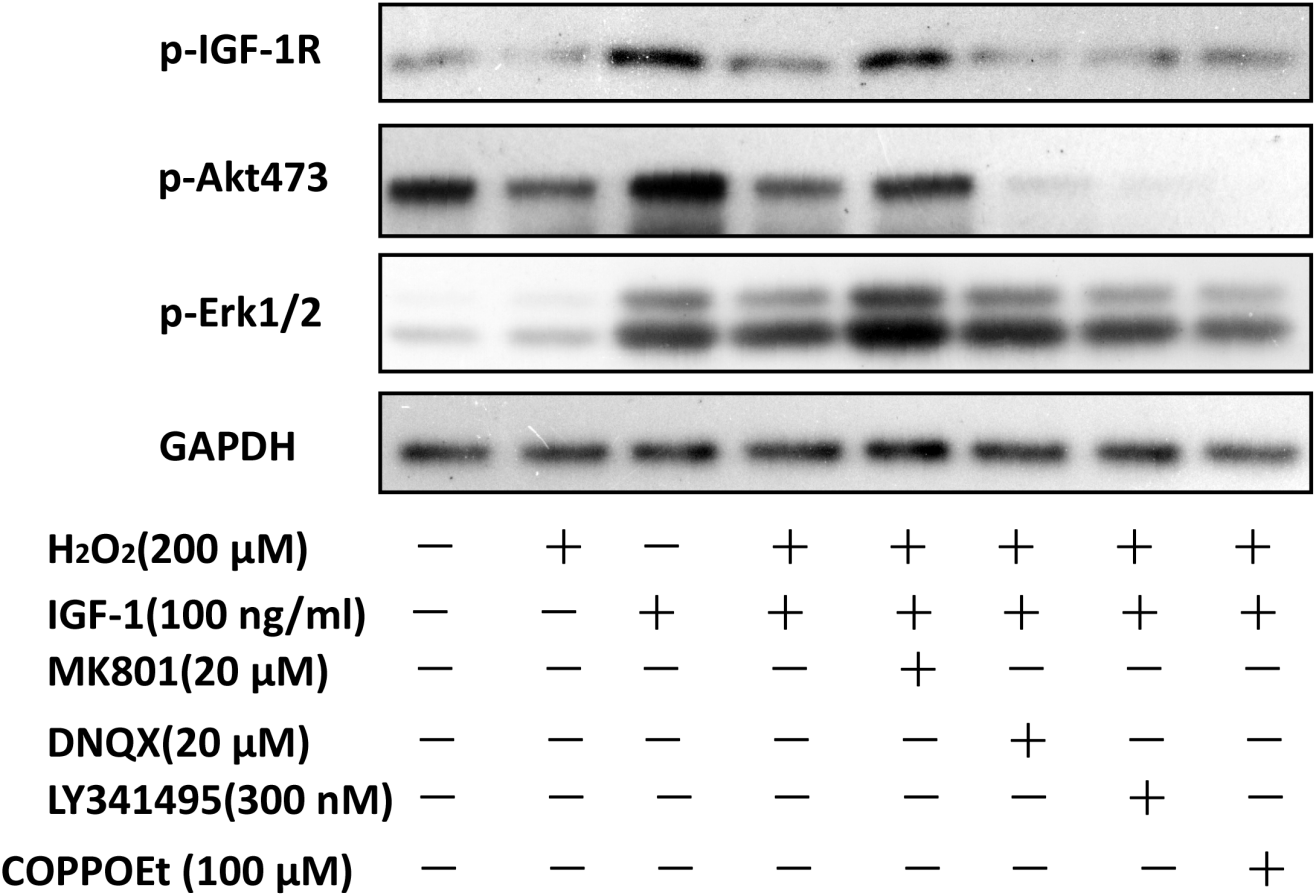

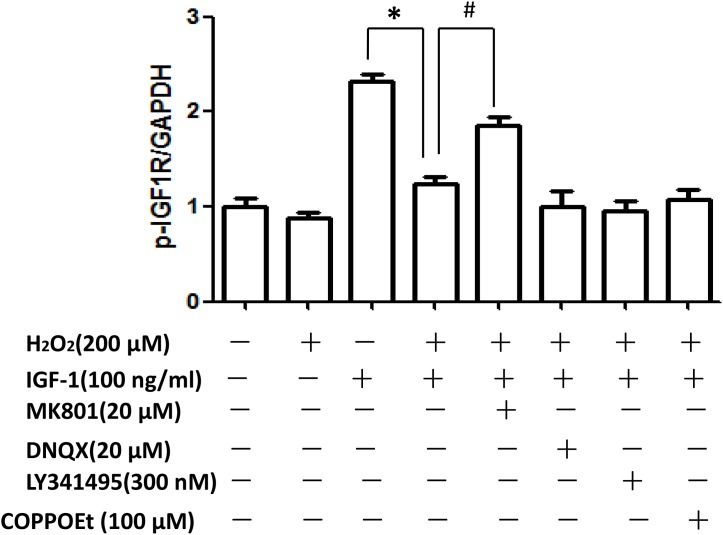
MK-801 restores IGF-1R, AKT and ERK1/2 phosphorylation stimulated by IGF-1 and the protective effect of IGF-1 in SH-SY5Y cells SH-SY5Y cells were treated with MK-801, DNQX, LY341495, or COPPOEt for 30min followed by a treatment of H_2_O_2_ (200μM) for another 60min, and then stimulated by IGF-1. **(a)** After 24h IGF-1 exposure, cell viabilities was determined by the MTT. **(b)** For protein study, cells were harvested after IGF-1 stimulation for 10 min. Phosphorylation of IGF-1R, AKT and ERK1/2 was analyzed by western blotting with specific antibodies as described. **(c)** Relative levels of p-IGF1R versus GAPDH was determined by densitometry of the blots. Densitometric analysis of the western blot was expressed as a percentage of control. Results are shown as the mean ± SEM and represent three independent experiments. *p <0.05 compared with IGF-1. #p <0.05 compared with H_2_O_2_+IGF-1.

As mentioned above, both IGF-1 neuroprotective properties and the phosphorylation of IGF-1R upon IGF-1 stimulation were attenuated by H_2_O_2_ in SH-SY5Ycells. We have reported previously that the activation of NMDA receptors impaired the survival signaling of IGF-1 in cultured hippocampal neurons [14]. Therefore, we tested the effects of glutamate receptor on the inhibitory effect of H_2_O_2_using various glutamate receptor antagonists [i.e. N-methyl-D-aspartate (NMDA) receptor antagonist MK-801, mGlu II/III antagonist LY341495, AMPA and kainate receptors antagonist DNQX, and noncompetitive mGlu I antagonist CPCCOEt]. Compared with H_2_O_2_+IGF-1, cell viability was observed to be significantly increasing after treatment with MK-801 (P<0.01; Figure 3a), whereas no significant change in cell viability was seen with DNQX, CPCCOEt, or LY341495 (P>0.05; Figure 3a). In parallel, the phosphorylation of IGF-1R, AKT and ERK1/2 were fully restored by MK-801 while DNQX, LY341495, and CPCCOEt had no effect (Figure 3b and 3c). These results suggested that in H_2_O_2_ induced toxicity is mediated by only NMDA receptors.

### NR2B inhibitor but not NR2A inhibitor abolished the inhibitory effect of H_2_O_2_ on the pro-survival signaling and effects of IGF-1 in SH-SY5Y cells

**Figure 4:**
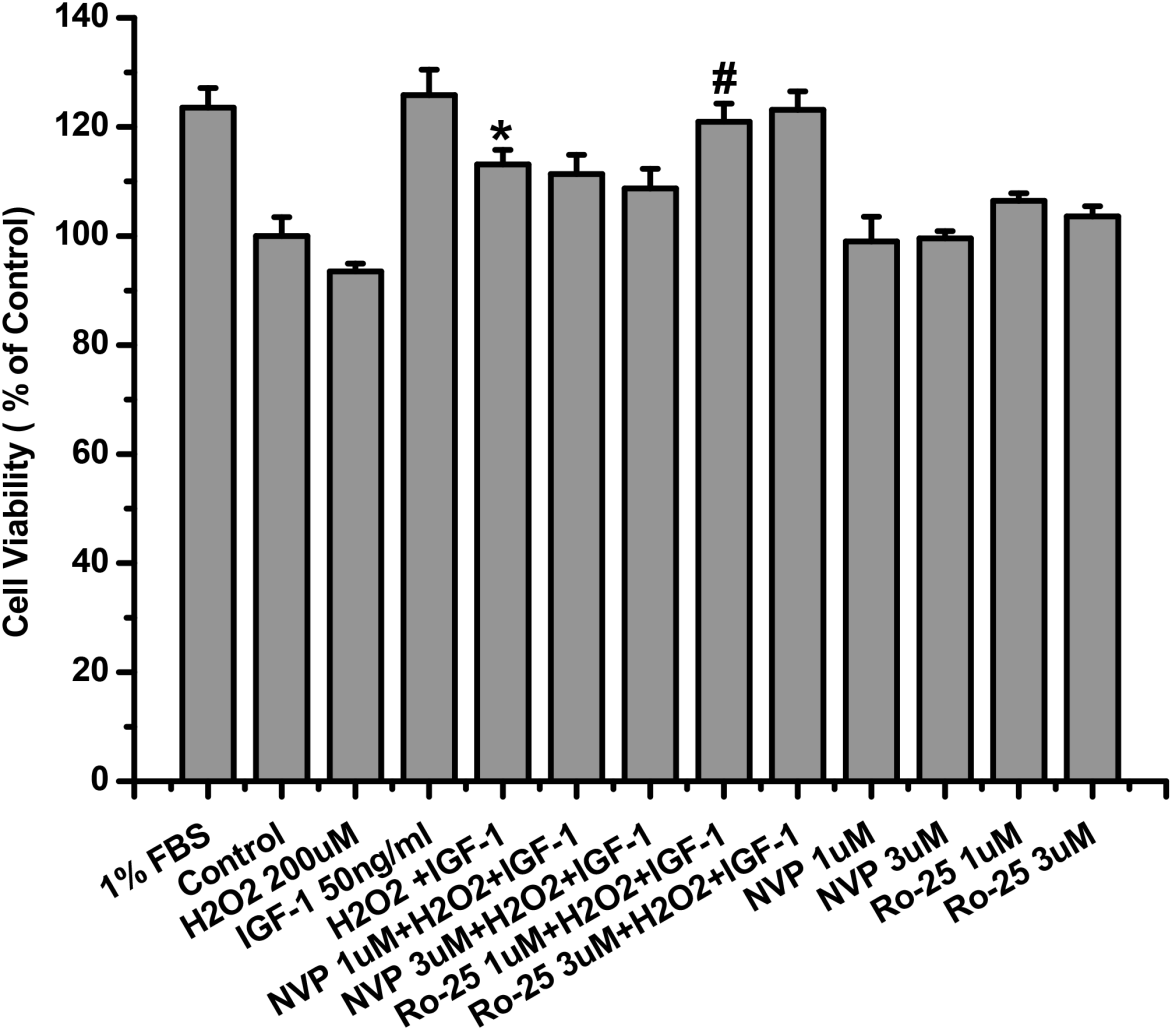

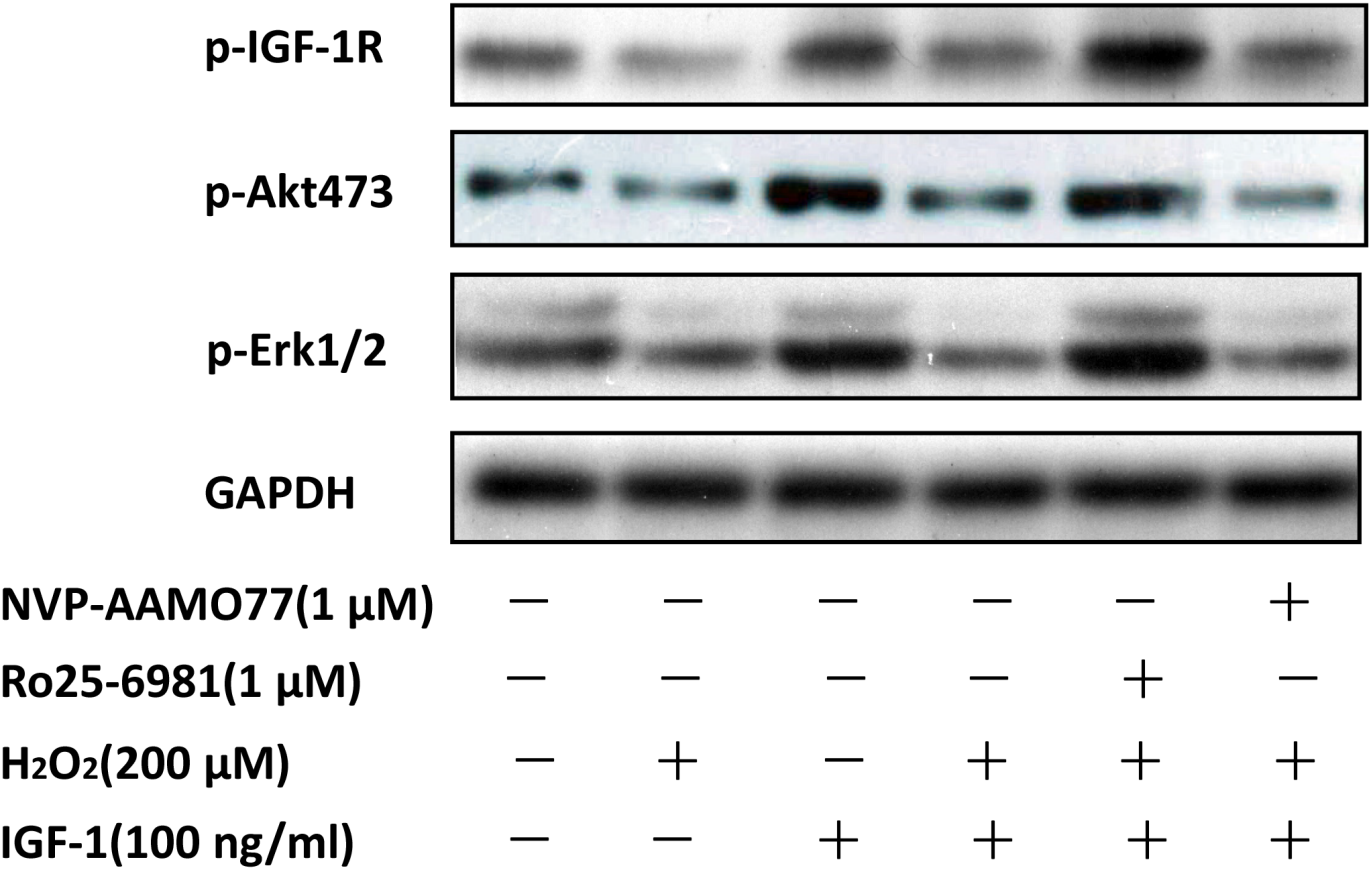

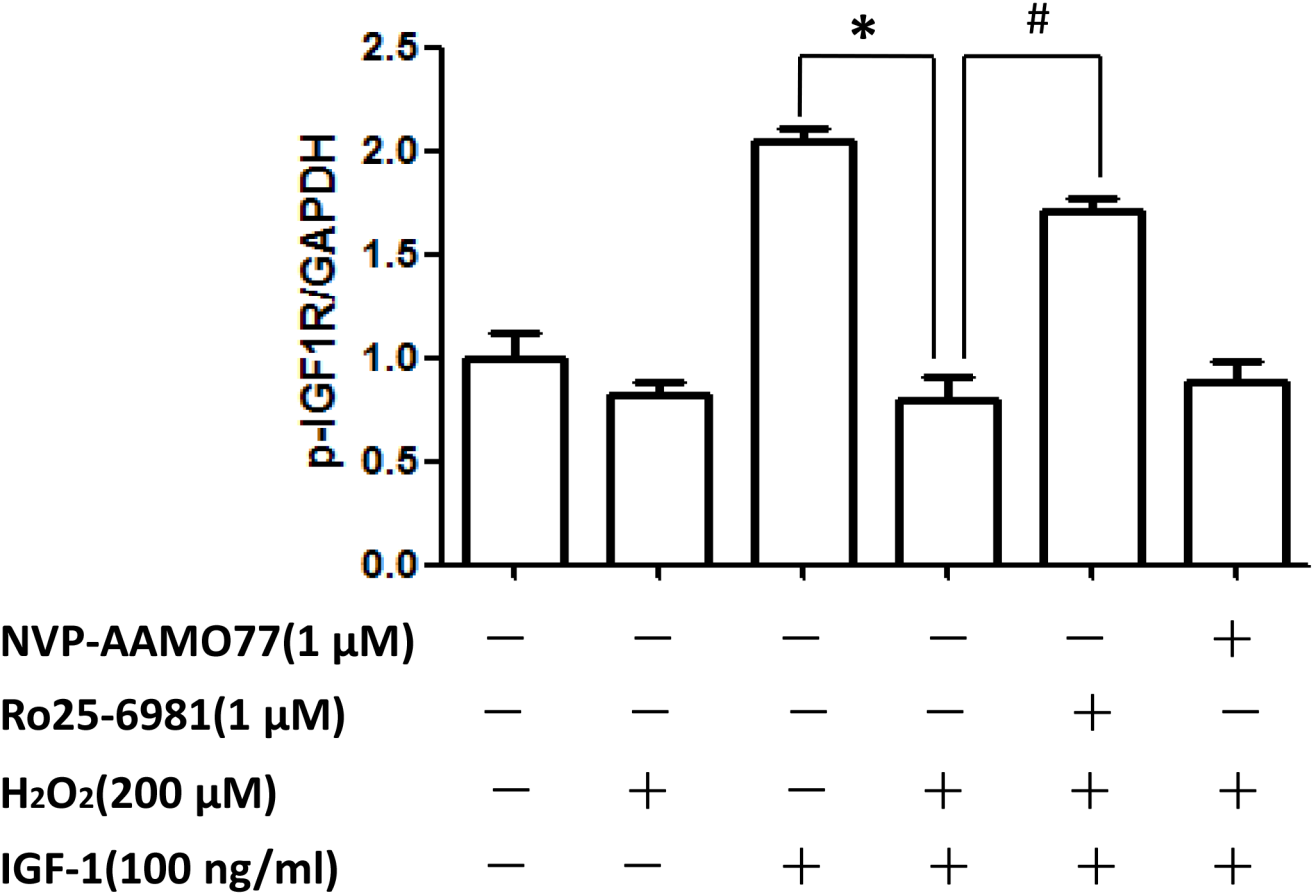
NR2B inhibitor abolished the inhibitory effect of H_2_O_2_ on the pro-survival signaling and effects of IGF-1 in SH-SY5Y cells SH-SY5Y cells were treated with NR2B containing NMDA receptor inhibitor Ro25-6981 or NR2A inhibitor NVP-AAMO77 for 30min, followed by a treatment of H_2_O_2_ (200μM) for 60min, and then stimulated by IGF-1. **(a)** 24h later, cell viabilities were measured by MTT. **(b)** For western blotting, cells was harvested after IGF-1 stimulation for 10 min. Expression of phosphorylated IGF-1R, AKT and ERK1/2 was analyzed by western blot. **(c)** Relative levels of p-IGF1R versus GAPDH was determined by densitometry of the blots. Densitometric analysis of the western blot was expressed as a percentage of control. Results are shown as the mean ± SEM and represent three independent experiments. *p <0.05 compared with IGF-1. #p <0.05 compared with H_2_O_2_+IGF-1.

Having proved that NMDA receptors were involved in the effects of H_2_O_2_ to inhibit the survival properties of IGF-1 in SH-SY5Ycells, we investigated which subunit of NMDA receptors mediated the effects of H_2_O_2_ by NMDA receptor subunit specific inhibitors. As shown in Figure 4a, NR2B containing NMDA receptor inhibitor Ro25-6981 rather than NR2A inhibitor NVP-AAMO77 reversed the effects of H_2_O_2_ on cell viability reduction upon IGF-1 stimulation in SH-SY5Ycells. Consistent with MTT results, western blot data also showed that Ro25-6981 reversed the inhibitory effect of H_2_O_2_ on the phosphorylation of IGF-1R and AKT and ERK1/2, whereas NR2A inhibitor NVP-AAMO77 had no effect (Figure 4b and 4c).

### siRNA NR2B knockdown but not that of NR2A reversed the inhibitory effect of H_2_O_2_ on the pro-survival signaling of IGF-1 in SH-SY5Y cells

**Figure 5:**
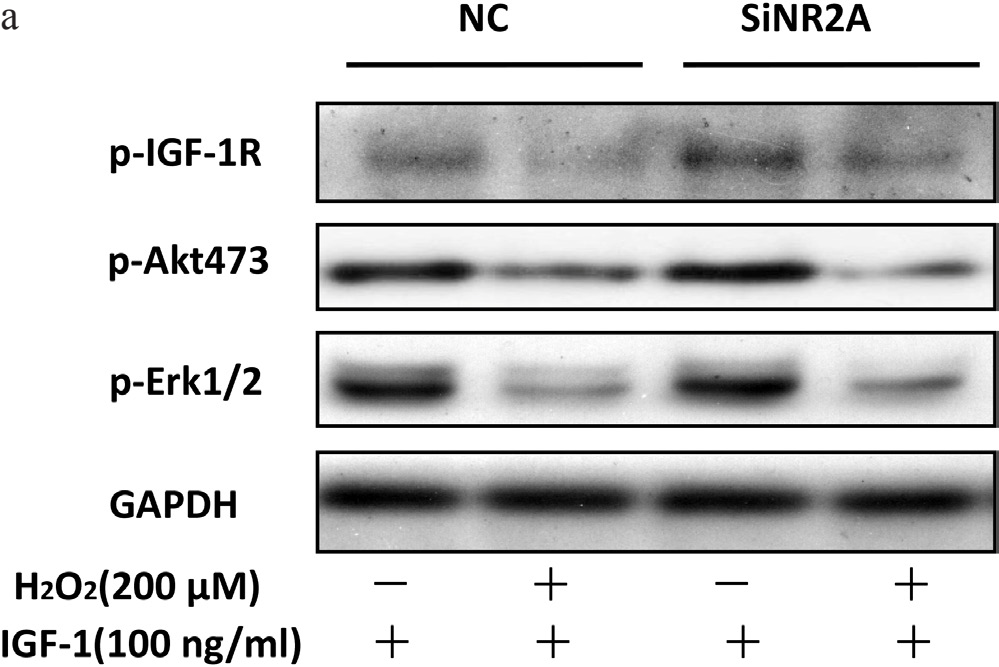

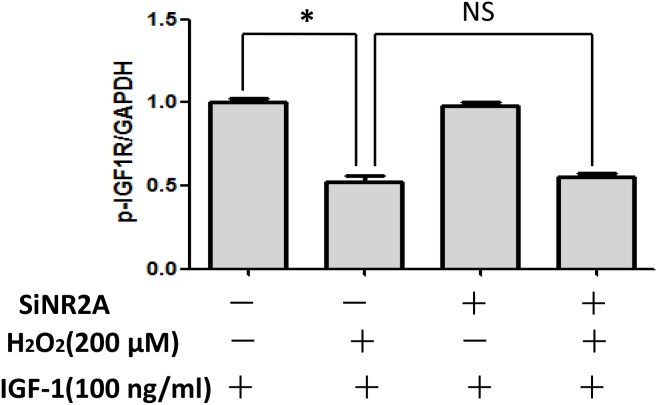

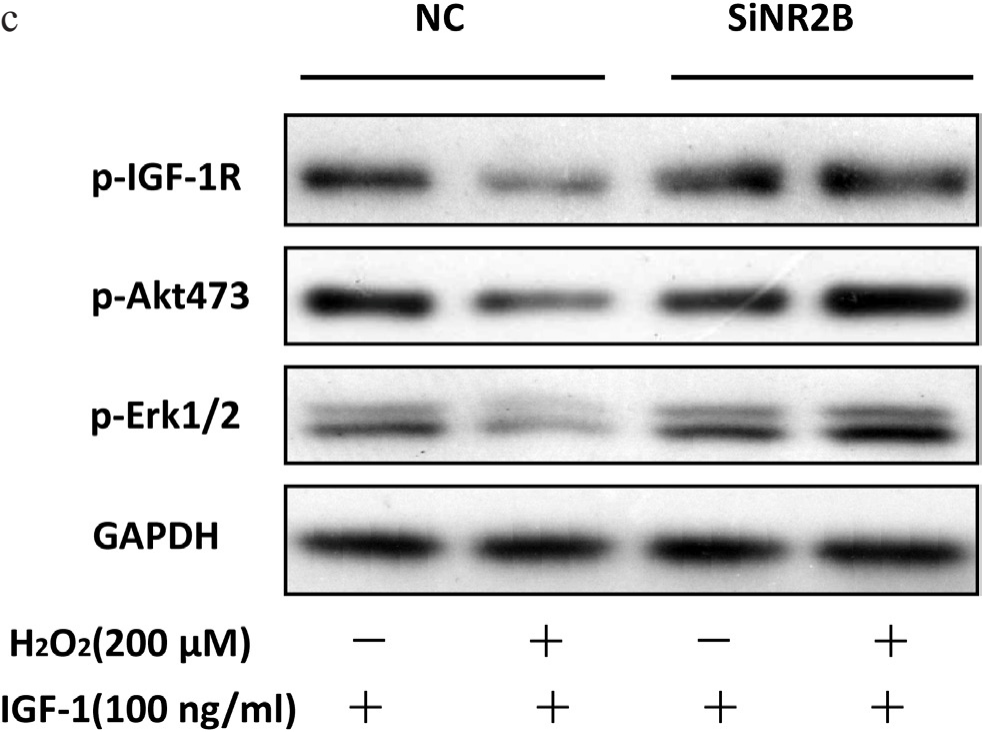

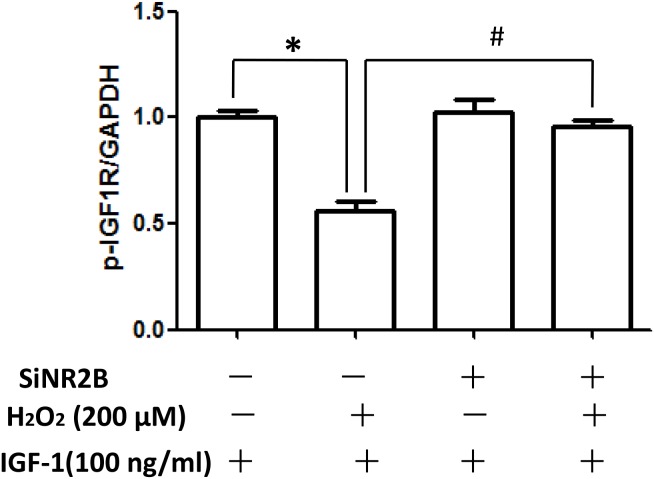
siRNA NR2B knockdown reversed the inhibitory effect of H_2_O_2_ on the pro-survival signaling of IGF-1 in SH-SY5Y cells **(a, c)** SH-SY5Y cells were transfected with specific siRNA for NR2A or NR2B, 48h later, these cells were exposure to H_2_O_2_ (200μM) for 60min, and then stimulated by IGF-1 for 10min. Expression of phosphorylated IGF-1R, AKT and ERK1/2 was analyzed by western blot. **(b, d)** Relative levels of p-IGF1R versus GAPDH was determined by densitometry of the blots. Densitometric analysis of the western blot was expressed as a percentage of control. Results are shown as the mean ± SEM and represent three independent experiments. *p <0.05 compared with control only stimulated with IGF-1. #p <0.05 compared with control siRNA.

To validate the role of the NR2B containing NMDA receptor in mediating the effects of H_2_O_2_, we transfected SH-SY5Y cells with specific siRNA to down regulate the NR2B or NR2A protein which leads to inhibition of NMDA receptors. Figure 5c and 5d showed that knockdown of NR2B significantly diminished the inhibitory actions of H_2_O_2_ on the phosphorylation of IGF-1R, AKT and ERK1/2 while NR2A knockdown had no effect (Figure 5a and 5b). These data together with data obtained with pharmacological inhibitors above verified that the NR2B containing NMDA receptor mediated the inhibitory effects of H_2_O_2_ on the protective effect of IGF-1.

### EDTA but not EGTA rescued the inhibitory effect of H_2_O_2_ on the pro-survival signaling of IGF-1 in SH-SY5Y cells

**Figure 6:**
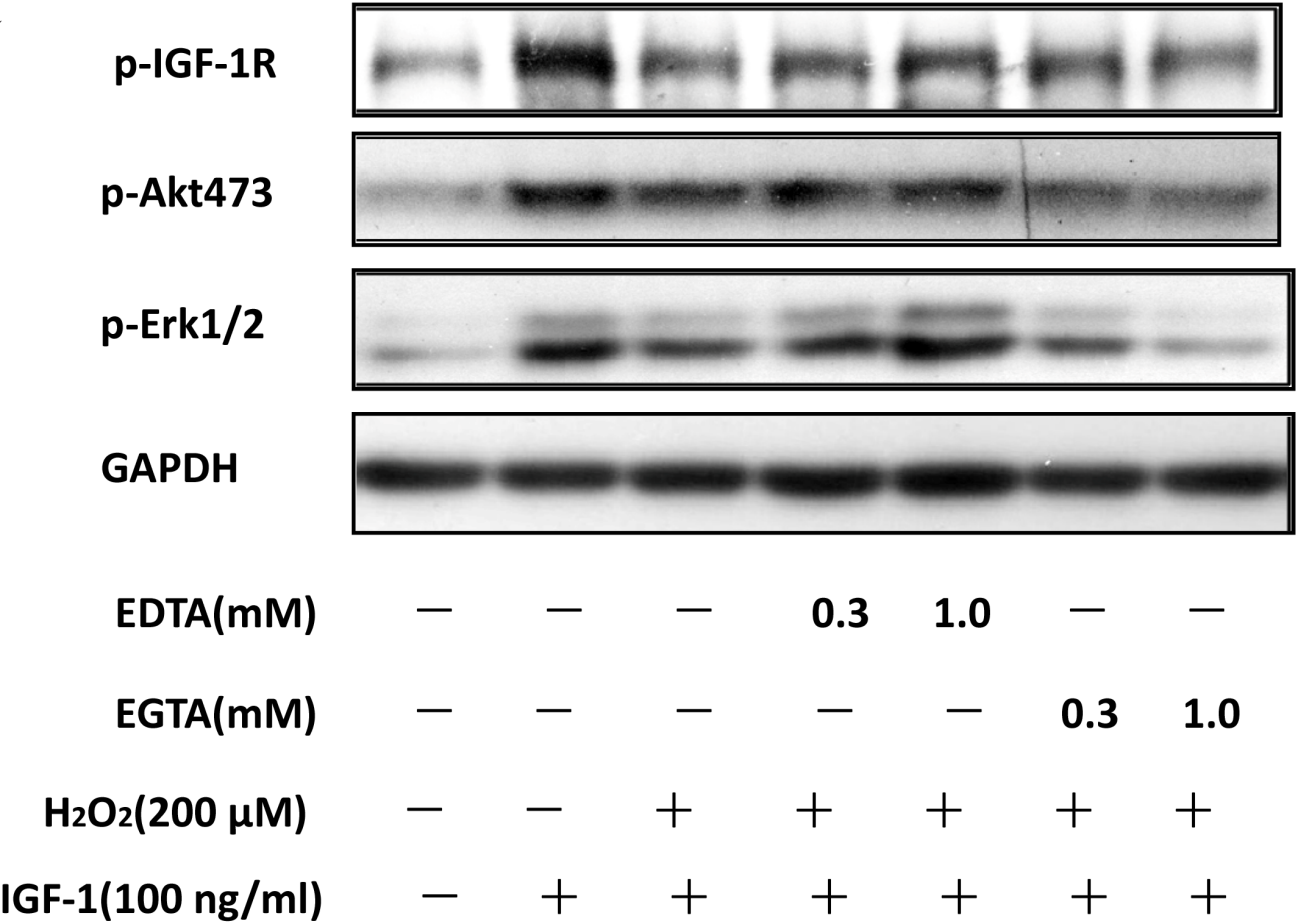

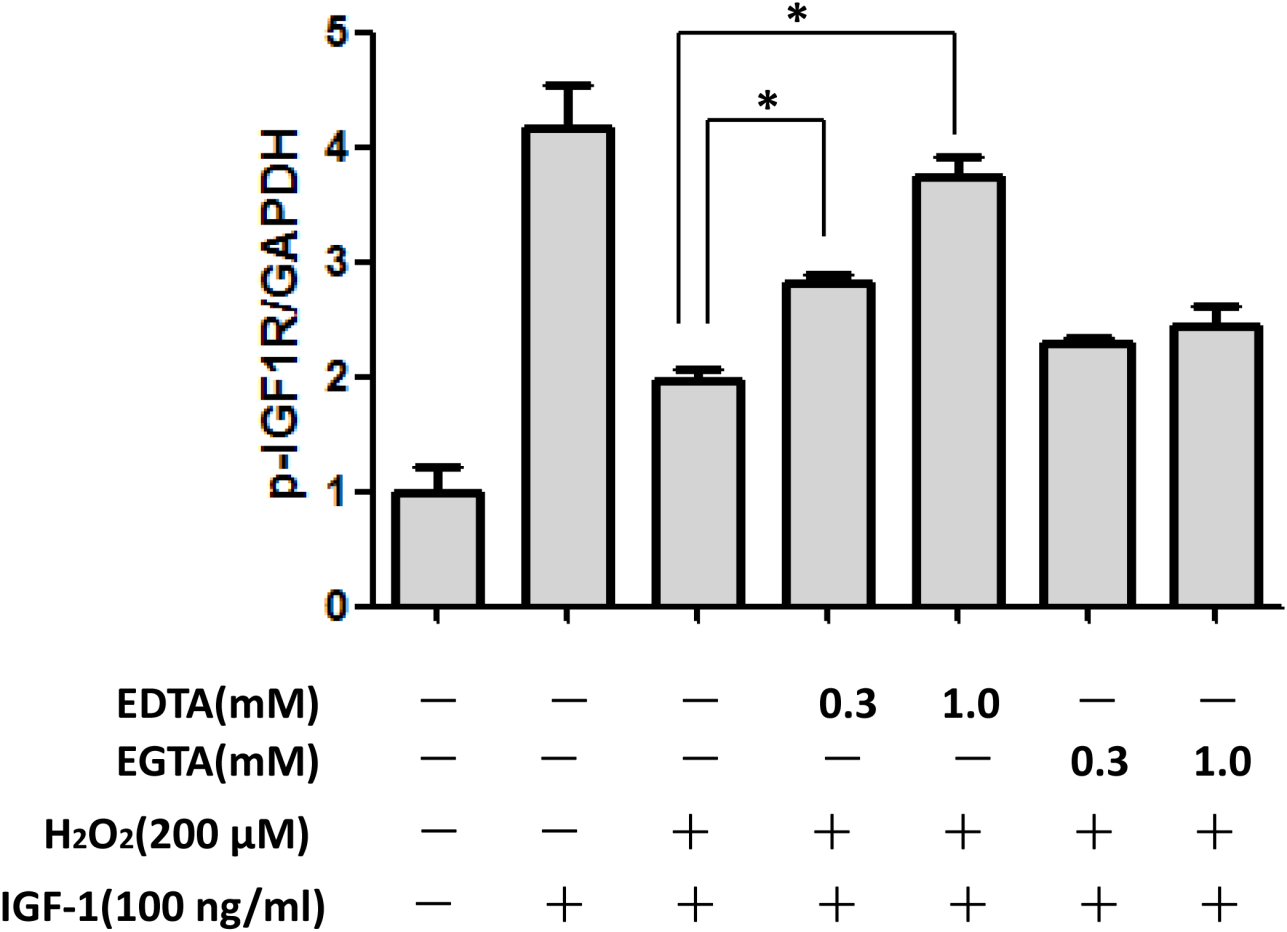
Extracellular Ca^2+^ chelation EDTA rescued the inhibitory effect of H_2_O_2_ on the pro-survival signaling of IGF-1 in SH-SY5Y cells SH-SY5Y cells were treated with Ca^2+^ chelation EDTA or EGTA for 30min, followed by a treatment of H_2_O_2_ (200μM) for 60min, and then stimulated by IGF-1for 10min. **(a)** Expression of phosphorylated IGF-1R, AKT and ERK1/2 was analyzed by western blot. **(b)** Relative levels of p-IGF1R versus GAPDH was determined by densitometry of the blots. Densitometric analysis of the western blot was expressed as a percentage of control. Results are shown as the mean ± SEM and represent three independent experiments. *p <0.05 compared with H_2_O_2_+IGF-1.

As Ca^2+^ influx is major risk event upon NR2B activation. Here, we explored whether Ca^2+^ influx resulted from NR2B containing NMDA receptor over activation contributes to the effects of H_2_O_2_. As shown in Figure 6, EDTA but not EGTA significantly reduced the inhibitory effects of H_2_O_2_ on the phosphorylation of IGF-1R, AKT and ERK1/2. These data suggested that Ca^2+^ influx also contributed to the inhibitory effects of H_2_O_2_ on the protective effect of IGF-1.

### H_2_O_2_ attenuated the pro-survival signaling and effects of IGF-1 in primary cultured cortical neurons via MADA receptor

**Figure 7:**
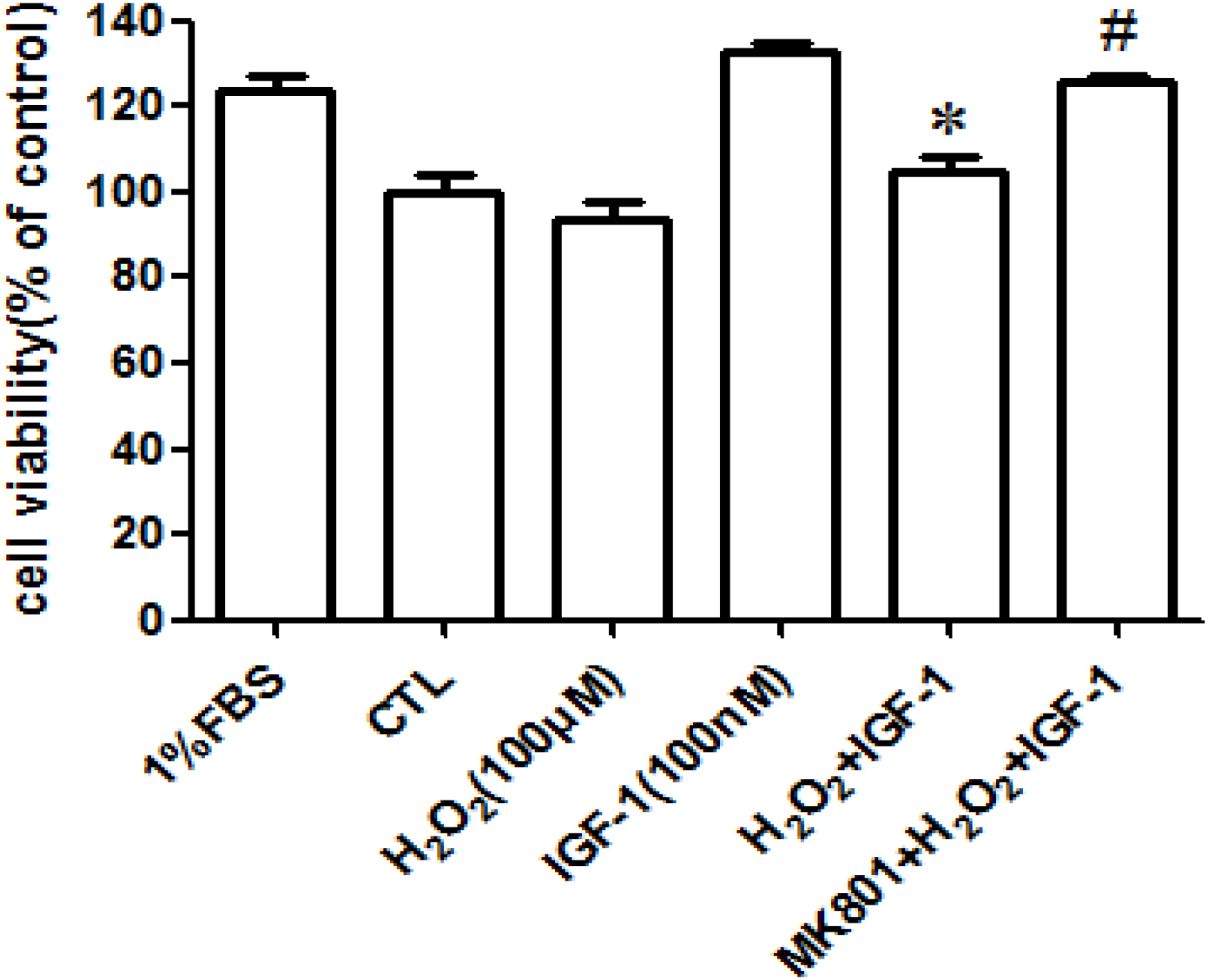

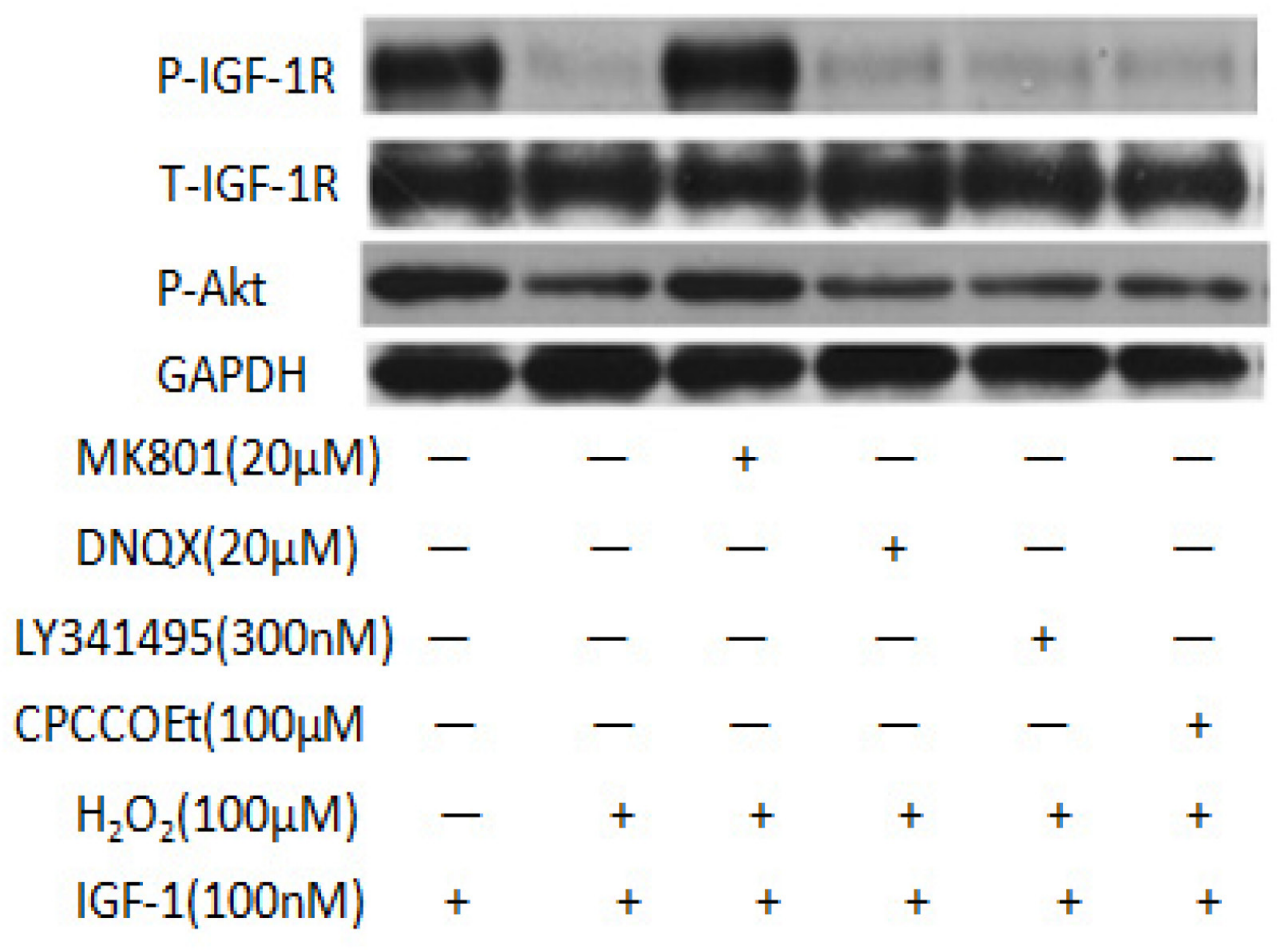
MK-801 restores IGF-1R, and AKT phosphorylation stimulated by IGF-1 and the protective effect of IGF-1 in primary cultured cortical neurons **(a)** Cortical neurons were treated with MK-801 for 30min followed by the treatment with H_2_O_2_ (100μM) for another 60min, and then stimulated by IGF-1 for 24h and the cell viabilities were measured by MTT. **(b)** For protein study, cortical neurons were treated with MK-801, DNQX, LY341495, or COPPOEt for 30min followed by a treatment of H_2_O_2_ (100μM) for another 60min, after IGF-1 stimulation for 10 min, cells were harvested. Phosphorylation of IGF-1R, and AKT were analyzed by western blotting with specific antibodies as described. *p <0.05 compared with IGF-1. #p <0.05 compared with H_2_O_2_+IGF-1.

After knowing that H_2_O_2_ attenuated the pro-survival signaling and survival effects of IGF-1 in SH-SY5Y cells via MADA receptor, we further validated these results in primary cultured cortical neurons. As expected, H_2_O_2_ inhibited the survival protection (Figure 7a) and the phosphorylation of IGF-1R and AKT (Figure 7b) induced by IGF-1 in primary cultured cortical neurons, preincubated cortical neurons with NMDA receptor antagonist reversed the inhibitory effect of H_2_O_2_. These results indicated that H_2_O_2_ inhibited IGF-1 pro-survival signaling and effects in primary cultured cortical neurons via MADA receptor (Figure 7) and are consistent with our results in SH-SY5Y cells.

## DISCUSSION

In present study, we explored the effect of H_2_O_2_ on the survival effect of IGF-1 in human SH-SY5Y cells and their underlying mechanisms. Our results showed that H_2_O_2_ blocked the survival effect and signaling of IGF-1 in these human neuronal cells. Further studies with specific glutamate receptor antagonists and siRNA indicated that the inhibitory effect of H_2_O_2_ is mediated by the NR2B containing NMDA receptor.

Oxidative stress, caused due to disparity between the production and removal of ROS, disturbs the pro-survival signaling leading to the development of neuronal diseases such as Parkinson’s disease (PD), Alzheimer’s disease (AD), and ischemic stroke [3, 31, 32]. IGF-1, as a polypeptide trophic factor is produced by nearly all cell types in the brain and with higher expression during perinatal stage and plays an important role in growth and development after birth [33]. In adult mammalian brain, IGF-1 is produced at much lower levels with a pattern defined expression surrounding the cortex, hippocampus, cerebellum, and spinal cord. In addition peripheral IGF-1 can also be produced by the liver [33]. Under some conditions, neuronal activity evoked by electrical, sensory, or behavioral stimulation has been shown to activate the transportation of IGF-1 to the CNS, promoting neuronal growth and survival [34]. Similarly, we found that IGF-1 promoted cell survival in human SH-SY5Y cells against serum deprivation. Meanwhile, the pro-survival signaling of IGF-1 was interfered by H_2_O_2_, and ultimately affected the viability of SH-SY5Y cells promoted by IGF-1 *in vitro*.

Mechanisms underlying the inhibitory effect of H_2_O_2_ on IGF-1 signaling in SY5Y is not known at present. We have previously shown that NMDA receptors play an essential role in attenuation of pro-survival of IGF-1 by glutamate in primary neurons [14, 19] while H_2_O_2_ was shown to induce extracellular accumulation of glutamate in neuronal cells. These findings support the possibility that H_2_O_2_ may inhibit the IGF-1 signaling by glutamate receptor pathway in SH-SY5Y cells. In support of this hypothesis, our data indicated that the inhibitory effect of H_2_O_2_ on the survival effect of IGF-1 in SH-SY5Y cells is mediated by NMDA receptor as only NMDA receptor antagonist MK801, but not other glutamate subtype inhibitors, blocked the effect of H_2_O_2_ in SH-SY5Y cells.

In mammalian CNS, Glutamate is the major excitatory transmitter which plays a pivotal role in excitatory synaptic transmission, neural development, and plasticity [35, 36]. N-methyl-D-aspartate (NMDA) receptors account for a major subtype of glutamate receptors at extra-synaptic sites which link multiple intracellular catabolic processes responsible for irreversible neuronal insult [37]. NMDA receptors are reported to play an essential role in attenuation of pro-survival of IGF-1 by glutamate in primary neurons [14, 19] and consistent with our present findings, it has a key role in the inhibitory effect of H_2_O_2_ in human neuronal cell Sh-SY5Y cells.

As mentioned before, two obligatory NR1 and two NR2A-D subunits constitute the heteromeric NMDARs complexes [38]. Both NR2A- and NR2B-containing NMDA receptors are regarded as the major types of functional NMDA receptor channels in CNS neurons because of the very low probability of channel opening in NR2C- (and possibly NR2D-) containing receptors [39, 40]. In earlier reports, it was suggested that the accumulation of glutamate due to oxidative stress resulted in the activation of NMDA receptors, most likely via the NR2B subunits [27]. Therefore, the activation of NMDA receptors induced by oxidative stress should be suppressed by NR2B inhibitor, and this can interrupt the IGF-1 signaling. As expected, NR2B inhibitor Ro25-6981 was able to restore the survival promoting effects and the phosphorylation of IGF-1R. We thus suggest that H_2_O_2_ impairs IGF-1 signaling through NR2B containing NMDA receptors. This speculation was further supported by the siRNA knockdown studies where NR2B knockdown was sufficient to reverse the effects of H_2_O_2_, and reduction of the NR2A expression had no effects.

The influx of Ca^2+^ plays central role in the sustained activation of NMDA receptors [41]. Under pathological conditions, Ca^2+^ overload leads to the excessive Ca^2+^ influx through the receptor channels, triggering multiple intracellular catabolic processes, and thereby inducing an irreversible insult of neuronal cells [42]. Further study was carried out to identify whether Ca^2+^ influx contributed to NMDA receptors activation by H_2_O_2_. The results revealed that blocking the Ca^2+^ influx by EDTA but not EGTA was sufficient to reverse the effects of H_2_O_2_. Thus, elevation of Ca^2+^ influx through extrasynaptic NMDA receptor channels due to oxidative stress contributes to the inhibition of the promoting survival effect of IGF-1.

To conclude with, this study revealed that H_2_O_2_, acting through the NR2B containing NMDA receptor, is capable of diminishing the IGF-1R signaling and survival effects in SH-SY5Y cells. These findings indicate that H_2_O_2_ can control neuronal viability by altering trophic factor receptor signaling which was implicated in neurodegenerative disease through NR2B. Therefore, NMDAR antagonists, especially NR2B-selective ones, combined with IGF-1 may serve as an alternative therapeutic agent for oxidative stress related neurodegenerative disease.

## MATERIALS AND METHODS

### Materials

Human SH-SY5Y cell line was obtained from cell bank, Sun Yat-sen University (Guangzhou, China). Human recombinant IGF-1 was obtained as a gift from Genentech Inc. (San Francisco, California, USA). Dulbecco’s modified Eagle’s medium (DMEM), and fetal bovine serum (FBS) were purchased from Gibco-BRL (NY, USA). BCA protein assay kit was obtained from Beyotime Institute of Biotechnology (Haimen, China); Anti-phospho-AKT (Ser473, Ser308) antibody, anti-AKT antibody, anti-phospho-p44/42 MAPK (ERK1/2) (Thr202/Tyr204) antibody, anti-ERK1/2 antibody were from Cell Signaling Technology (Woburn, USA); Anti-phospho-IGF-1R (Tyr1135/1136) and anti-IGF-1R (1135/1136) were purchased from Signalway Antibody Co. Ltd (College Park, Maryland, USA). Anti-GAPDH was purchased from Proteintech Group Inc. (Chicago, Illinois, USA). 6,7-Dinitroquinoxaline-2,3-dione (DNQX), CPCCOEt, Ro25-6981, NVP-AAMO77and LY341495 were purchased from Tocris Bioscience Inc. (Minneapolis, Minnesota, USA). H_2_O_2_ (30%), dizocilpine maleate (MK-801), 3-(4,5-Dimethylthiazol-2-yl)-2,5-diphenyl tetrazolium bromide (MTT), EDTA, EGTA and DMSO were from Sigma Aldrich (St Louis, Missouri, USA).

### Cell culture

SH-SY5Y cells were cultured in high-glucose Dulbecco’s modified Eagle’s medium (DMEM) containing 5 % fetal bovine serum (FBS), 100μg/ml streptomycin, 100 U/ml penicillin and incubated at 37°C with 5% CO_2_ humidified atmosphere. Cultured media was replaced twice a week with fresh medium as described above. Stock culture was routinely sub-cultured at 1:4 ratio at a weekly interval.

### MTT assay

Cell viability was assessed by using a 3-(4,5-Dimethylthiazol-2-yl)-2, 5-diphenyl- tetrazolium bromide (MTT) assay as described previously [29]. Briefly, cells were seeded in 96-well plates at a density of 2×10^5^ cells/well with 100μl 1% FBS. After 12-16h, the culture medium was replaced with 90μl of fresh DMEM. The cultures were incubated with H_2_O_2_ and presence or absence with individual inhibitors for 60 min, then exposed to IGF-1(50ng/ml). 24h later MTT assay was performed. Then the cells were incubated with MTT (0.5 mg/ml) for additional 3 h. The medium was aspirated from each well and DMSO (150μl) (Sigma, USA) was added. The absorbance of each well solution was obtained with a Multiskan Ascent Revelation Plate Reader (Thermo, USA) and the data are presented as Optical Density (OD) at wavelength of 570nm. Assays were repeated at least three to six times.

### Western blot analysis

Western blot analysis was performed as previously described [30]. Briefly, cells from different experimental conditions were lysed with ice-cold RIPA lysis buffer, and protein concentration was determined with a BCA protein assay kit according to the manufacturer’s instructions. Equal amounts of lysate protein were subjected to SDS-PAGE with 10 % polyacrylamide gels and electrophoretically transferred to nitrocellulose membranes. Nitrocellulose blots were first blocked with 4 % bovine serum albumin (BSA) in PBST buffer (PBS with 0.01 % Tween 20, pH 7.4), and incubated overnight at 4 °C with primary antibodies in PBST containing 1% BSA. Immunoreactivity was detected by sequential incubation with horseradish peroxidase-conjugated secondary antibodies, and then detected by the enhanced chemiluminescence technique.

### Transfection of small interfering RNA (siRNA)

SH-SY5Y cells were transfected using the Amaxa nucleofection technology. Briefly, cells were subjected to centrifugation and resuspended in Amaxa nucleofector buffer solution, following the Amaxa guidelines for cell line transfection that we have optimized for SH-SY5Y cells. For siRNA transfection, SH-SY5Y cells (2.5×10^5^) in 25 μl of buffer solution (20.5 μl Rat Neuron Nucleofector Solution+4.5 μl supplement) were mixed with 50 pmol negative control siRNA or siRNA specific for NR2A or NR2B (GenPharma Co, Ltd, Shanghai, China). Cells were then immediately nucleofected with an Amaxa nucleofector apparatus (program: RAW264.7high viability; Amaxa), and after transfection, cells were immediately transferred to complete medium and cultured in 24-well plates at 37 °C.

### Data analysis and statistics

Data are expressed as mean±SEM or mean±SD. Variation between groups was analyzed using oneway ANOVA, which was followed by Student–Newman–Keuls or Dunnett’s T3 procedures when the assumption of equal variances did not hold. p Value <0.05 was considered statistically significant. Statistical analyses were conducted with SPSS 13.0.

## Abbreviations

AD: Alzheimer’s disease
AKT: protein kinase B
CNS: central nervous system
DNQX: 6,7-dinitroquinoxaline-2,3-dione
EDTA: ethylenediaminetetraacetic acid
EGTA: ethylene glycol tetraacetic acid
ERK1/2: extracellular regulated protein kinases 1/2
H_2_O_2_: hydrogen peroxide
IGF-1: insulin-like growth factor-1
NMDA: N-methyl-D-aspartate
MAPK: mitogen-activated protein kinase
MK-801: dizocilpine maleate
PI3K: phosphatidylinositol 3-kinase
RNS: reactive nitrogen species
ROS: reactive oxygen species
SNP: sodium nitroprusside
